# Impact of Different Extraction Methods on Furanosesquiterpenoids Content and Antibacterial Activity of *Commiphora myrrha* Resin

**DOI:** 10.1155/2021/5525173

**Published:** 2021-07-10

**Authors:** Ali S. Alqahtani, Rashed N. Herqash, Omar M. Noman, Md. Tabish Rehman, Abdelaaty A. Shahat, Mohamed F. Alajmi, Fahd A. Nasr

**Affiliations:** Department of Pharmacognosy, College of Pharmacy, King Saud University, P. O. Box 2457, Riyadh 11451, Saudi Arabia

## Abstract

The oleo-gum-resin of *Commiphora myrrha* is one of the most known natural antimicrobial agents, mainly due to its furanosesquiterpenes. A validated method based on sample extraction by matrix solid-phase dispersion (MSPD) followed by high-performance column chromatography (HPLC) determination is applied to analyze two furanosesquiterpenoids, namely, 2-methoxyfuranodiene (CM-1) and 2-acetoxyfuranodiene (CM-2), existing in *C. myrrha*. The trial parameters that controlled the extraction prospective were studied and optimized. These include the nature of dispersant, mass ratio of sample to the dispersant, and the volume of elution solvent. A comparative antimicrobial study that used the Minimum Inhibitory Concentration Assay (MIC) method between MSPD, ultrasonic, and Soxhlet of myrrh extracts was also conducted. The optimal MSPD parameters used were (i) 15 mL of methanol applied as elution solvent; (ii) silica gel/sample mass at a 2 : 1 ratio; and (iii) a dispersing sorbent selected as silica gel. Technique retrievals were regulated from 96.87% to 100.54%, with relative standard deviations (RSDs) from 1.24% to 4.45%. *Commiphora myrrha*-MSPD (CM-MSPD) extract showed the highest antibacterial activity against gram-positive and gram-negative bacteria (156.25 *μ*g/mL and 312.5 *μ*g/mL, respectively) and antifungal activity (156.25 *μ*g/mL). Yields acquired through the MSPD technique were larger than yields from other extraction techniques (sonication and traditional reflux extraction methods) with less consumption of time, sample, and solvent. The mode of antibacterial action of CM-1 and CM-2 was elucidated by performing molecular docking with bacterial DNA gyrase. Both the compounds interacted with key residues of DNA gyrase.

## 1. Introduction

The medicinal plant *Commiphora myrrha* (family Burseraceae) produces the aromatic oleo-gum-resin, known as myrrh. The genus *Commiphora* include over 150 species of trees and shrubs located mainly in Africa, India, Yemen, and the southern regions of Saudi Arabia. [1]. Hundreds of phytochemicals of myrrh were identified and examined for various therapeutic activities since the plant was discovered [2]. The composition of myrrh is 30%–60% gum (including acidic polysaccharides), 25%–40% resin, and 3%–8% volatile oil (eugenol, herbolene, and many furanosesquiterpenes) [3]. Two furanosesquiterpenoids, 2-methoxyfuranodiene (CM1) and 2-acetoxyfuranodiene (CM2), were previously isolated and identified from the ethanolic extract of myrrh [4]. Using cytotoxic MTT assay, both compounds displayed a promising activity against liver (HepG2) and breast (MCF-7) cancer cells with IC_50_ values 3.6 and 4.4 *μ*m, respectively [5]. Moreover, the influence of variations of 2-methoxyfuranodiene and 2-acetoxyfuranodiene content on the biological properties of seventeen commercial samples of *Commiphora myrrha* showed variability in furanosesquiterpenoids content (CM-1 and CM-2) with highest antioxidant activity for samples collected from Saudi Arabia [4]. Therefore, simple, effective, and rapid techniques are necessary for the extraction of these two major phytochemical constituents (i.e., 2-methoxyfuranodiene and 2-acetoxyfuranodiene). Barker et al. presented solid-phase dispersion matrix (MSPD) innovation as a new technique to extricate medical entities from animal tissue [6]. Since then, the MSPD technique has garnered widespread intense interest as it conducts extraction and cleaning in a single step and successfully fractionates the semisolid, solid, and highly sticky samples [7]. MSPD has been recently used as an alternative to customary extraction techniques for extricating constituents from therapeutic plants [7–11]. According to the literature review, there is no available trial on MSPD as a type of extraction of 2-methoxyfuranodiene and 2-acetoxyfuranodiene from *C. myrrha* that we currently know of. Zhang et al. as well as AlZain et al.'s methods of extraction were followed and adopted [12, 13].

## 2. Materials and Methods

### 2.1. Chemicals and Reagents

**Figure 1:**
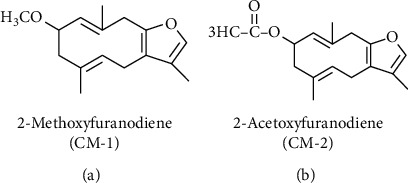
Structures of the two furanosesquiterpenes compounds. (a) 2-Methoxyfuranodiene (CM-1). (b) 2-Acetoxyfuranodiene (CM-2).

2-Methoxyfuranodiene (CM1) and 2-acetoxyfuranodiene (CM2) were selected as standards. The chemical structures of these compounds are shown in Figure 1.

### 2.2. Plant Material

The oleo-gum-resin of *C. myrrha* used was purchased from the Alothaim Market in Riyadh, Saudi Arabia. Five 100 g samples of the grinded gum resin were used for the experiments.

### 2.3. HPLC Instrumentation and Chromatographic Conditions

For quantification, an Alliance 2695 Separations Module equipped with 2487 dual wavelength absorbance detectors (Waters Instruments, Inc., Milford, MA, USA) was used. Chromatographic analysis was carried out using the following equipment: built-in quaternary pump; Pinnacle C18 column (5 *μ*m, 250 × 4.6 mm); four-channel degasser; and an autosampler with programmable temperature (25°C). The mobile phase was composed of different proportions of 0.5% formic acid in ultrapure water (A), and a 1 : 1 mixture of acetonitrile and methanol (B) was used with a flow rate of 1 mL/min. The optimized gradient program was as follows:

- 0–16 min (10%–30% B)
- 16–17 min (30%–40% B)
- 17–20 min (40%–60% B)
- 20–22 min (60%–95% B)
- 22–23 min (95%–85% B)
- 23–25 min (85%–10% B)
- 25–30 min (10% B)

Samples were filtered by PDVF 0.45 *μ*m syringe filter and then injected into the system at 20 *μ*L. The output signal (254 nm) was detected and processed using EMPOWER software, version 2.

### 2.4. MSPD Extraction Method

A *C. myrrha* powder and silica gel (100 and 200 mg, resp.) were put together and mixed for 5 min to obtain a homogeneous uniformity. Thereafter, the mixture was transferred into glass syringe (5 mL) preloaded with cotton at the bottom to serve as an adsorbent layer. To prevent any contamination and spillage, a second piece of cotton was attached on the top of the mixture. Eluent collection was performed in a volumetric flask which was filled up with MeOH to a level of 15 mL. A filter (0.45 *μ*m) was used to filter the obtained extract which was subsequently analyzed by HPLC.

### 2.5. Soxhlet Extraction Method

Since centuries, Soxhlet extraction represents the standard method for effective extraction among the diverse extraction procedures [14, 15]. Around 500 mg of a *C. myrrha* was packed in a thimble and then put on Soxhlet extractor. Under reflux, methanol (90 mL) was then added into a distillation flask and heated for 6 h. To a 100 mL volumetric flask the extract was gently transferred and filled up with CH_3_OH. Before the HPLC analysis, the extract solution was filtered through a 0.45 *μ*m filter.

### 2.6. Sonication Extraction Method

The *C. myrrha* sonication extraction was achieved by weighting 250 mg of sample, placed into a 20 mL volumetric flask and extracted with 30 mL of methanol. Thereafter, the mixture was subjected to sonication for 20 min. Sample solution was then filtered through a 0.45 *μ*m filter before HPLC analysis.

## 3. Biological Studies

### 3.1. Determination of the Antimicrobial Activity

#### 3.1.1. Test Microorganisms

Four bacteria species including two gram-positive (*Staphylococcus aureus* and ATCC 25923; *Enterococcus faecalis* and ATCC 29212) and two gram-negative (*Escherichia coli* and ATCC 25922; *Proteus vulgaris* and ATCC 8427), as well as one *Candida albicans* (ATCC 60193) fungal strain were used in this investigation.

#### 3.1.2. Minimum Inhibitory Concentrations

MICs for methanolic extracts of *C. myrrha* resulting from the three extraction s (CM-Sonication, CM-Soxhlet, and CM-MSPD) were tested for their antimicrobial activity as described by Mann and Markham [16]. Twofold serial dilutions of each extract (100 *μ*L/well) were pipetted to 96-well culture plates. Both extracts were made in the required broth media to obtain 2,000 to 31.2 mg/mL concentrations. Suspension of 100 *μ*L and 1,106 CFU/mL bacteria and fungi was then added and incubated at the appropriate temperatures for 24 h and 72 h, respectively. The lowest concentration displaying no detectable bacterial or fungal growth (MIC) was determined. Five microliters from the wells showed no growth and was transferred to agar plates. They were then incubated for 24 h or 72 h for minimum bactericidal/fungicidal concentrations (MBC/MFC). Both gentamycin and nystatin were used as positive controls.

### 3.2. Molecular Docking

The binding and interaction of CM-1 and CM-2 with bacterial DNA gyrase B was evaluated by molecular docking using AutoDock4.2 as described earlier by Al-Shabib et al. [17]. 2D structures of CM-1 and CM-2 were drawn on ChemSketch. PyRx v0.8 was used to minimize the energies of CM-1 and CM-2 employing the universal force field (UFF). The minimized states of CM-1 and CM-2 were converted into ready-to-dock pdbqt format in PyRx v0.8. The three-dimensional coordinates of bacterial DNA gyrase B (PDB ID: 4KFG) were downloaded from RCSB databank and preprocessed before conducting molecular docking. The water molecules and all bound ligands were removed, H-atoms were added, and bond orders were defined in the structure of DNA gyrase B. A fresh network of H-bonds was defined, and the energy of the complete system was minimized using UFF [18]. Molecular docking was performed inside a 22.5 × 17.5 × 20.7 Å dimension grid box centered at 30.2 × 18.5 × −2.3 Å with 0.375 Å spacing. Lamarckian Genetic Algorithm combined with Solis and Wets local search methods were utilized for molecular docking, as described previously by Rabbani et al. [14]. The initial positions of CM-1 and CM-2, their orientations, and torsions were set indiscriminately. A total of 10 docking runs were enumerated. For each run, a maximum of 2,500,000 energy terms were calculated. The population size was set to 150, and the translational step was fixed at 0.2 Å. The torsions and quaternions were set at 5. The results were analyzed, and final images were prepared in Discovery Studio (Accelrys). Docking affinities (*K*_*d*_) of CM-1 and CM-2 for the kinase domain of DNA gyrase B were determined from docking energies (△*G*) using the following equation [15]:

(1) $$\begin{array}{c} △G=\;-RT\;\ln\;K_{d}, \end{array}$$

where *R* and *T* represent Boltzmann gas constant and temperature, respectively.

## 4. Results and Discussion

### 4.1. Optimization of MSPD Procedure

MSPD is a widely used and effective extraction technique that comprises the extraction, destruction, and purifying functions in a single step. So, as to obtain the maximal extraction yield of the two furanosesquiterpene compounds from *C. myrrh*, the most convenient extraction factors from the MSPD process were optimized. The experiments were carried out to regulate the essential factors, such as the ratio of scattering sorbent to sample, sort of scattering sorbent, and the volume of the eluting dissolvable which eventually determine the extraction yield of the final concentrate.

#### 4.1.1. Selection of Dispersing Sorbent

**Figure 2:**
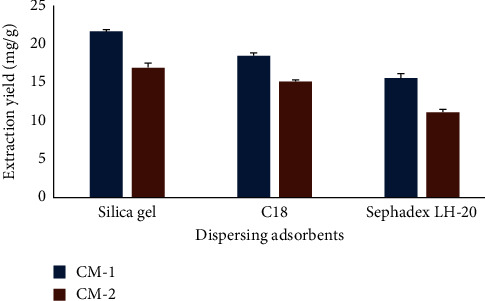
Effect of the dispersing sorbents on extraction yields.

Sorbent dispersion acts as both a bound and rough solvent. It disintegrates the sample matrix, disturbs its components, and elevates the interaction between solvent and sample through the MSPD mixing procedure. Therefore, the specific selectivity of an MSPD method is entirely dependent on the sorbent applied [19]. Three dispersing solvents were attempted in this phase, comprising silica gel, C18, and Sephadex LH-20.  Figure 2 illustrates CM-1 and CM-2 extraction yields from *C. myrrha* acquired from the three different dispersing solvents. The extraction yields of CM-1 and CM-2 were higher when using silica gel than the yields of extraction with C18. Hence, silica gel was selected as scattering sorbent designated for the MSPD method as it provided the finest extraction production of the two furanosesquiterpenes (i.e., 2-methoxyfuranodiene and 2-acetoxyfuranodiene) and was comparatively cheaper to purchase.

#### 4.1.2. Effect of the Sample Sorbent Mass Ratio

**Figure 3:**
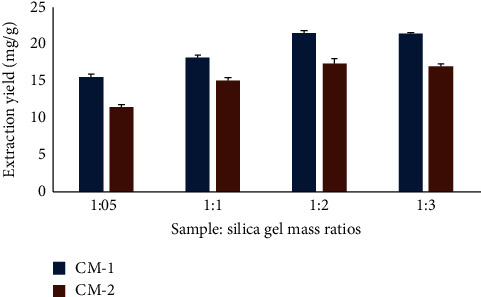
Effect of the sample sorbent mass ratio.

A balanced ratio of scattering sorbent to sample could build the contact zone among test and scattering sorbents and could improve the adsorption of examinations on scattering sorbents. Because of this, four different sample-to-silica gel mass ratios were studied, ranging from 0.5 : 1 to 1 : 3. These results are displayed in Figure 3. The 1 : 2 sample-to-silica gel mass ratio resulted in the highest extraction yield for the 2 furanosesquiterpenes. However, a further increase in the mass ratio to 1 : 3 resulted in a reduction in the extraction yields of both compounds. Thus, in this work, 1 : 2 was selected as the optimal mass ratio.

#### 4.1.3. Effect of Elution Solvent Volume

**Figure 4:**
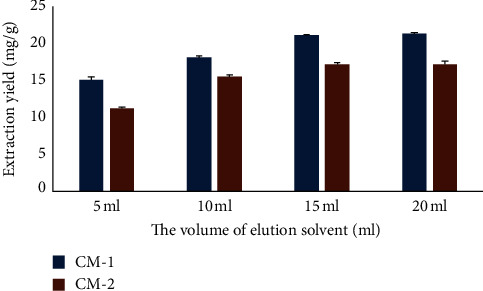
Effect of elution volume on extraction yields.

When executing an MSPD method, the elution volume is an important factor to consider. The extraction procedure was carried out using four different volumes of organic solvent methanol (5, 10, 15, and 20 mL). Results (Figure 4) demonstrated that the yields of CM-1 and CM-2 increased with increasing methanol volumes from 5 mL to 15 mL; the yields were fixed after the final increase to 20 mL. Therefore, 15 mL was selected to ensure complete desorbing of the compounds from dispersing sorbents at the minimum solvent consumption.

### 4.2. MSPD-HPLC Analysis

**Figure 5:**
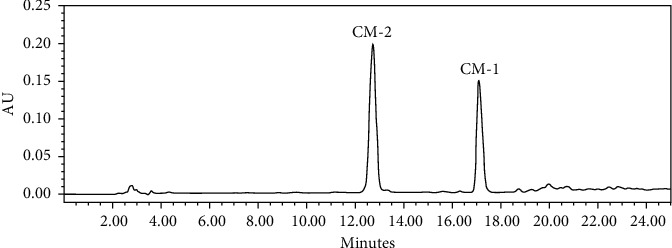
HPLC chromatograms of standard of two furanodiene compounds.

The combined standard solutions of HPLC chromatograms from *C. myrrha* extracts are depicted in Figure 5. The developed method was validated to establish the fact that its performance features were compatible with the desired performance for analyzing the two *C. myrrha* sample compounds. Validation of the methods was performed based on the guidelines set by the International Conference on Harmonization [20]. The following validation features were evaluated: linearity, quantitation limit of the analytes, repeatability of the results (precision), and recovery.

Linearity was evaluated by building external calibration curves for each compound using a working standard solution containing both compounds. Calibration curves were studied based on the linear correlation between the analyte peak area (*y*-axis) and its concentration (*μ*g/mL), for six different concentration levels (0.5, 1.0, 2.0, 5.0, 12.00, and 25.00 *μ*g/mL). Each concentration of the mixed standard solution was injected in triplicate, and regression parameters were calculated. The estimated coefficients of correlation (*r*^2^) of calibration curves were better than 0.996, thus proving the linearity of the proposed quantitative method (Table 1).

Sensitivity of the method was assessed by calculating the limits of detection (LOD) and quantification (LOQ) of the two analytes. LOD and LOQ were calculated based on the calibration curve and calculated according to examples (1) and (2):

1. LOD = 3.3 *σ*/*S*
2. LOQ = 10 *σ*/*S*

where *σ* is the standard deviation of the response and *S* is the slope of the calibration curve.

The LOD of the developed method was 0.60 and 0.62 *μ*g/mL for CM-1 and CM-2, respectively, and the corresponding LOQ values were 1.82 and 1.87 *μ*g/mL (Table 1). Precision of the developed method was determined based on two parameters: repeatability of the results and intermediate precision. The repeatability was assessed based on results of the replicate analysis (*n* = 3) carried out within the same day (intraday). The intermediate precision was evaluated according to the results of consecutive analyses (*n* = 3) carried out over three days (interday). Repeatability and intermediate precision of the methods were expressed as relative standard deviation (RSD). The %RSD in the repeatability test was in the range of 0.28%–3.91% for CM-1 and 0.29%–1.05% for CM-2, respectively. The corresponding intermediate precision ranges were 0.36%–4.00% and 0.45%–1.15% (Table 2). The assay provided satisfactory results as the overall %RSD values for both intra- and interday tests were less than 4.00%, which indicates that the developed method was in accordance with required specifications.

Accuracy of the developed method was assessed by means of a recovery experiment; the peak areas obtained from *C. myrrha* samples previously fortified with known quantities of standard analytes (CM-1 and CM-2) were compared with blank “unfortified” samples at three different concentration levels (high, medium, and low). The percentage recoveries were calculated based on the following equation: (total determined amount × original amount)/added amount × 100%.  Table 3 shows mean recoveries of analyzed compounds within the range of 95.13%–100.39% with RSD less than 1.41%, indicating that the developed method is accurate enough for the quantification of both *C. myrrha* compounds.

### 4.3. Antimicrobial Study

The antimicrobial effects resulting from the *C. myrrha* three extraction methods (CM-Sonication, CM-Soxhlet, and CM-MSPD) in terms of MIC and MBC/MFC are displayed in Table 4. CM-MSPD exhibited stronger antimicrobial activity than CM-Sonication and CM-Soxhlet extracts; the most sensitive strains are the gram-positive *Staphylococcus aureus* and *Enterococcus faecalis* (*E. faecalis*; MIC: 156.25 mg/mL). The MBC or MFC values were approximately two times higher than the MICs (Table 4). This could be in response to the presence of a high content of phytochemical constituents, including 2-methoxyfuranodiene and 2-acetoxyfuranodiene.

### 4.4. Comparison of MSPD, Soxhlet, and Sonication Procedures

**Figure 6:**
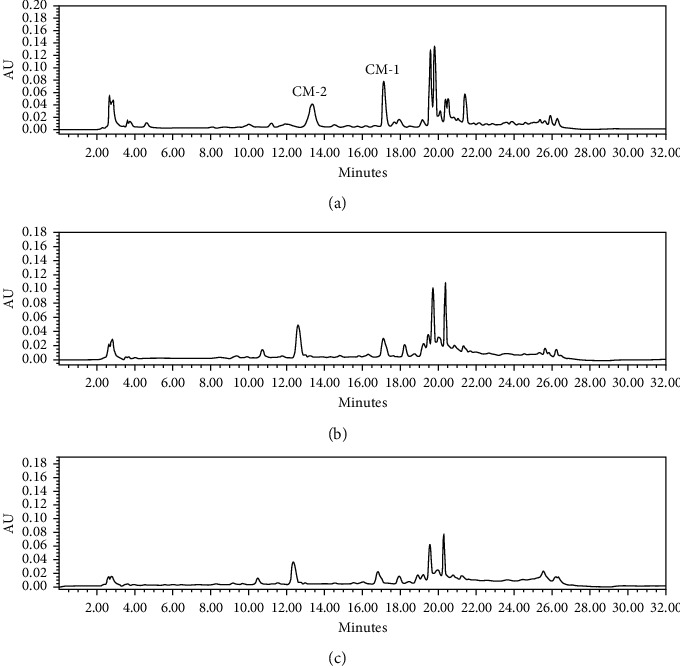
HPLC chromatograms of *C. myrrh.* (a) MSPD extraction method. (b) Soxhlet extraction method. (c) Sonication extraction method.

Table 5 shows the comparison of MSPD, Soxhlet, and sonication extraction methods. Results showed that MSPD showed a maximum extraction yield of the two furanosesquiterpenes compounds (38.7 mg/g) as compared to the Soxhlet (33.75 mg/g) and extraction method (29.3 mg/g) (Figure 6). The MSPD method also required only a 0.1 g sample, 15 mL solvent, and 15 minutes to extract the target furanosesquiterpenes from *C. myrrha*. This demonstrated that solvent, sample, and time consumption were decreased by the MSPD method compared to conventional extraction methods like Soxhlet, sonication, and other new techniques. More importantly, the extraction of MSPD does not require heating. Ultimately, the MSPD method as an instrumental criteria tool is beneficial. Implementing the method only requires an affordable cartridge and mortar that could be created by any chemical laboratory [13]. These benefits show that the extraction of MSPD from *C. myrrh* should be an appropriate method for extracting target furanosesquiterpenes.

### 4.5. Molecular Docking Analysis

**Figure 7:**
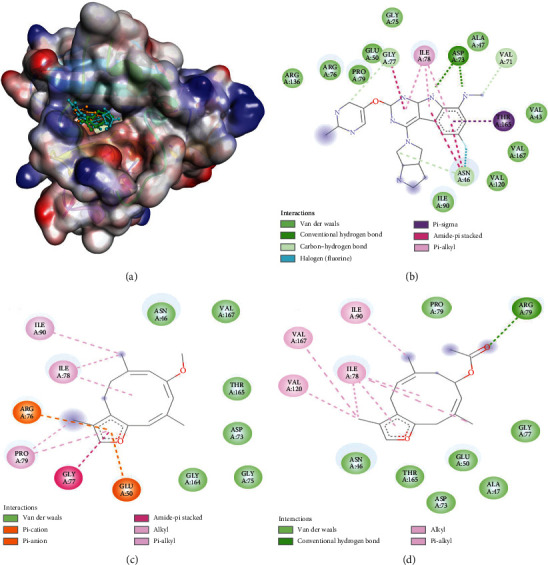
Molecular docking and interaction between DNA gyrase B and control ligand, CM-1 and CM-2. (a) Binding of control ligand (golden color), CM-1 (teal color), and CM-2 (green color) at the ATP binding domain of DNA gyrase B. Interaction between DNA gyrase B and control ligand (b), CM-1 (c), and CM-2 (d).

Bacterial DNA gyrase is a type II topoisomerase which can bind DNA and introduce negative supercoils during replication at the expense of ATP hydrolysis. It is found in all bacteria and is absent from higher eukaryotes, thus serving as a potential target to design antibacterial agents. It contains two subunits, A and B, which associate together to form the A2B2 complex in the active enzyme form. The A subunit of DNA gyrase binds DNA and harbors active-site Tyr residue for the cleavage, while subunit B of DNA gyrase harbors a kinase domain where ATP hydrolysis takes place. In this study, the ability of furanosesquiterpenes (CM-1 and CM-2) to bind the kinase domain of DNA gyrase using molecular docking was evaluated. The docking of CM-1 and CM-2 with DNA gyrase B has resulted in multiple low-energy poses. Binding poses with the lowest energy was further investigated for detailed protein-ligand interactions (Figure 7 and Table 6). It was evident that CM-1 and CM-2 were bound to the ATP binding site of DNA gyrase B. CM-1 formed 2 electrostatic interactions with Arg76: NH1 and Glu50: OE1; and 7 hydrophobic interactions with Arg76, Gly77, Ile78, Pro79, and Ile90. Some other residues forming van der Waals' interaction with CM-1 were Asn76, Gly75, Asp73, Gly164, Thr165, and Val167. The docking energy and the corresponding binding affinity of CM-1 for DNA gyrase B were estimated as −7.5 kcal mol^−1^ and 3.17 × 10^5^ m^−1^, respectively. Conversely, CM-2 interacted with DNA gyrase B through a conventional hydrogen bond with Arg76: HH12, and 6 hydrophobic interactions with Ile78, Ile90, and Val167. The residues Asn46, Ala47, Glu50, Asp73, Gly77, Pro79, and Thr165 were involved in the formation of van der Waals' interactions with CM-2.

The docking energy of CM-2-DNA gyrase B interaction was determined as −7.9 kcal m^−1^, while the binding affinity of CM-2 for DNA gyrase B was 6.23 × 105 m^−1^. For comparative analyses, docking between cognate ligand (as a control) was performed and presented in an X-ray crystal structure with DNA gyrase B (Figure 7 and Table 5). The control ligand formed 2 conventional hydrogen bonds with Asp73: OD1 and 3 carbon hydrogen bonds with Asn46: OD1, Val71: O, and Gly77: O. Moreover, a halogen bond between F ion and Asn46: CG was also observed between the control ligand and DNA gyrase B. Further, the control ligand-DNA gyrase B complex was stabilized by 7 hydrophobic interactions with Asn46: C, O-Ala47: N, Gly77: C, O-Ile78: N, Ile78, and Thr165: CG2. Some other residues such as Val43, Ala47, Glu50, Gly75, Arg76, Pro79, Ile90, Val120, Arg136, and Val120 were evaluated. The docking energy and binding affinity of control ligands for DNA gyrase B were determined to be −9.3 kcal m^−1^ and 9.3 × 106 m^−1^, respectively.

It is evident that CM-1 and CM-2 were bound to a DNA gyrase B site where control ligands bind. The amino acid resides commonly occupied by CM-1 and control ligand were Glu50, Asp73, Gly75, Arg76, Gly77, Ile78, Pro79, Ile90, Thr165, and Val167, while the amino acid residue commonly occupied by CM-2 and control ligand were Ala47, Glu50, Asp73, Arg76, Gly77, Ile78, Pro79, Ile90, Val120, Thr165, and Val167. Previous studies have reported that rutin also occupied the same binding site on DNA gyrase B and interacted through Asn46, Ala47, Asp49, Glu50, Ala53, Asp73, Gly75, Arg76, Gly77, Ile78, Pro79, Ile90, Val118, Gly119, Val120, Arg136, and Thr165 [21]. In the DNA gyrase B crystallographic structure, Asp73 forms a hydrogen bond with the adenosine amino group of AMP and the mutation of Asp73 to Ala73 or Asn73 completely abolished the ATPase and DNA supercoiling activities [22]. Similarly, Gly77 is in close vicinity of the adenine ring (∼4.3 Å from C-2 carbon and ∼5.1 Å from N-3). Thus, any mutation in Gly77 has a dramatic effect on the ATP binding probability, which leads to reduced ATP hydrolysis activity. Another important residue, Ile78, lies ∼4.5 Å above the adenine ring and plays a significant role in ATP binding and its resulting hydrolysis. Thr165 is a key conserving amino acid residue in all DNA gyrase B subunits and type II topoisomerases. It also plays a crucial role in the binding of ATP [22].

## 5. Conclusions

MSPD was successfully utilized and followed by HPLC for the extraction and determination of two furanosesquiterpenes in *C. myrrha* in order to obtain the maximum yield. The properties of the adjusted essential factors comprising solvent volume, dispersing sorbent, and dispersing ratio of sorbent to sample on the MSPD extraction competence of furanosesquiterpenes have been mainly assessed and optimized. The overall authentication for the HPLC method was then determined, including quantification of the two furanosesquiterpene compounds, linearity, sensitivity limits, precisions, and recovery. Furthermore, the extraction yields obtained by the MSPD technique were compared with those produced by customary ultrasonic and Soxhlet techniques.

## Figures and Tables

**Table 1:** Calibration parameters and sensitivity data for two compounds using the proposed HPLC method.

Compound	Retention time (min)	Range (*μ*g/mL)	Linearity (*r*^2^)	LOD (*μ*g/mL)	LOQ (*μ*g/mL)
CM-1	17.126 ± 0.03	0.5–25.00	0.9965	0.60	1.82
CM-2	13.382 ± 0.05	0.5–25.00	0.9963	0.62	1.87

**Table 2:** Analytical results of repeatability and intermediate precision for two compounds in the *C. myrrha* sample.

Analyte	Conc. (*μ*g/mL)	Intraday^a^ (% RSD) (*n* = 3)	Interday^b^ (% RSD) (*n* = 3)
CM-1	0.5	0.28	1.30
	5	3.91	4.00
	25	0.81	0.36

CM-2	0.5	0.29	0.45
	5	4.01	0.93
	25	1.05	1.15

^a^Repeatability. ^b^Intermediate precision.

**Table 3:** Analytical recovery data of the two analysts during quantification (*n* = 6).

Analyte	C_added_ (*μ*g/mL)	Mean recovery (%)	RSD (%) (*n* = 6)
CM-1	0.5	97.11	0.47
	5	98.24	0.36
	25	96.43	0.79

CM-2	0.5	98.34	1.23
	5	95.13	1.41
	25	100.39	0.89

**Table 4:** Minimal inhibitory concentrations, minimal bactericidal concentrations (MBC), and minimal fungicidal concentrations (MFC) of the crude extracts of *C. myrrha*.

	Activity	*S. aureus*	*E. faecalis*	*E. coli*	*P. vulgaris*	*C. albicans*
CM-sonication	MIC	312.5	312.5	625	625	156.25
	MBC	625	625	1250	1250	—
	MFC	NT	NT	NT	NT	312.5

CM-Soxhlet	MIC	312.5	312.5	625	625	156.25
	MBC	625	625	1250	1250	—
	MFC	NT	NT	NT	NT	312.5

CM-MSPD	MIC	156.25	156.25	625	625	156.25
	MBC	312.5	312.5	1250	1250	—
	MFC	NT	NT	NT	NT	312.5

Gentamycin	MIC	7.8	7.8	3.9	3.9	NT
	MBC	15.6	15.6	7.8	7.8	NT

Nystatin	MIC	NT	NT	NT	NT	3.5
	MFC	—	—	—	—	7.0

**Table 5:** Comparison of MSPD with other extraction methods for the extraction of main furanosesquiterpenoids from *C. myrrha*.

	MSPD	Soxhlet	Sonication
Total extraction yield (mg/g)	38.7	33.75	29.3
Sample (g)	0.1	0.5	0.25
Solvent	15 mL MeOH	100 mL MeOH	30 mL MeOH
Time	15 min	3.5 h	0.5 h
Special apparatus	None	Soxhlet	Ultrasonicator

**Table 6:** Molecular docking parameters for the interaction of CM-1 and CM-1 with the ATP binding domain of bacterial DNA Gyrase B.

Donor-acceptor pair	Distance (Å)	Nature of interaction	Docking energy (kcal m^−1^)	Binding affinity, *K*_*d*_ (m^−1^)
*Control*				
LIG:H–ASP73:OD1	1.9066	Conventional H-bond	−9.3	6.62 × 10^6^
LIG:H–ASP73:OD1	1.9014	Conventional H-bond		
LIG:C–GLY77:O	3.3299	Carbon H-bond		
LIG:C–ASN46:OD1	3.5462	Carbon H-bond		
LIG:C–VAL71:O	3.6145	Carbon H-bond		
ASN46:CG–LIG:F	3.6104	Halogen bond		
THR165:CG2–LIG	3.8251	Hydrophobic (pi-sigma)		
ASN46:C, O; ALA47:N–LIG	4.4879	Hydrophobic (amide-pi)		
ASN46:C, O; ALA47:N–LIG	4.5389	Hydrophobic (amide-pi)		
GLY77:C, O; ILE78:N–LIG	4.7710	Hydrophobic (amide-pi)		
LIG–ILE78	5.1181	Hydrophobic (pi-alkyl)		
LIG–ILE78	4.6140	Hydrophobic (pi-alkyl)		
LIG–ILE78	4.3432	Hydrophobic (pi-alkyl)		

CM-1				
ARG76:NH1–LIG	4.0868	Electrostatic (pi-cation)	−7.5	3.17 × 10^5^
GLU50:OE1–LIG	4.3017	Electrostatic (pi-anion)		
GLY77:C, O; ILE78:N–LIG	4.4654	Hydrophobic (amide-pi)		
ILE78–LIG	4.0246	Hydrophobic (alkyl)		
LIG:C–ILE78	4.5502	Hydrophobic (alkyl)		
LIG:C–ILE90	3.8908	Hydrophobic (alkyl)		
LIG:C–PRO79	4.3208	Hydrophobic (alkyl)		
LIG–ARG76	5.0625	Hydrophobic (pi-alkyl)		
LIG–PRO79	4.6901	Hydrophobic (pi-alkyl)		

CM-2				
ARG76:HH12–LIG:O	2.6933	Conventional H-bond	−7.9	6.23 × 10^5^
ILE78–LIG	4.1178	Hydrophobic (alkyl)		
LIG:C–ILE90	4.1266	Hydrophobic (alkyl)		
LIG:C–ILE78	5.0569	Hydrophobic (alkyl)		
LIG:C–ILE78	5.1356	Hydrophobic (alkyl)		
LIG:C–VAL167	4.6831	Hydrophobic (alkyl)		
LIG–ILE78	5.0433	Hydrophobic (pi-alkyl)		

## Data Availability

All the data related to these findings are included in the manuscript.
